# Preliminary comparative genomics revealed pathogenic potential and international spread of *Staphylococcus argenteus*

**DOI:** 10.1186/s12864-017-4149-9

**Published:** 2017-10-23

**Authors:** Dao-Feng Zhang, Xiao-Yang Zhi, Jing Zhang, George C. Paoli, Yan Cui, Chunlei Shi, Xianming Shi

**Affiliations:** 1 MOST-USDA Joint Research Center for Food Safety, School of Agriculture and Biology & State Key Laboratory of Microbial Metabolism, Shanghai Jiao Tong University, Shanghai, 200240 China; 2 Yunnan Institute of Microbiology, School of Life Sciences, Yunnan University, Kunming, 650091 China; 3 USDA-MOST Joint Research Center for Food Safety & Molecular Characterization of Foodborne Pathogens Research Unit, U.S. Department of Agriculture, Agricultural Research Service, Eastern Regional Research Center, Wyndmoor, PA 19038 USA; 4 Present address: No. 800 Dongchuan RD. Minhang District, Shanghai, 200240 China

**Keywords:** *Staphylococcus aureus*, *Staphylococcus argenteus*, *Staphylococcus schweitzeri*, Comparative genomics, Virulence gene, Capsular polysaccharides, *Agr*, Biogeographical structure

## Abstract

**Background:**

*Staphylococcus argenteus* and *S. schweitzeri*, were recently proposed as novel species within *S. aureus* complex (SAC). *S. argenteus* has been reported in many countries and can threaten human health. *S. schweitzeri* has not been associated with human infections, but has been isolated from non-human primates. Questions regarding the evolution of pathogenicity of these two species will remain elusive until an exploratory evolutionary framework is established.

**Results:**

We present genomic comparison analysis among members of SAC based on a pan-genome definition, which included 15 *S. argenteus* genomes (five newly sequenced), six *S. schweitzeri* genomes and 30 divergent *S. aureus* genomes. The three species had divergent core genomes and rare interspecific recombination was observed among the core genes. However, some subtypes of staphylococcal cassette chromosome *mec* (SCC*mec*) elements and prophages were present in different species. Of 111 tested virulence genes of *S. aureus*, 85 and 86 homologous genes were found in *S. argenteus* and *S. schweitzeri*, respectively. There was no difference in virulence gene content among the three species, but the sequence of most core virulence genes was divergent. Analysis of the *agr* locus and the genes in the capsular polysaccharides biosynthetic operon revealed that they both diverged before the speciation of SAC members. Furthermore, the widespread geographic distribution of *S. argenteus*, sequence type 2250, showed ambiguous biogeographical structure among geographically isolated populations, demonstrating an international spread of this pathogen.

**Conclusions:**

*S. argenteus* has spread among several countries, and invasive infections and persistent carriage may be not limited to currently reported regions. *S. argenteus* probably had undergone a recent host adaption and can cause human infections with a similar pathogenic potential.

**Electronic supplementary material:**

The online version of this article (10.1186/s12864-017-4149-9) contains supplementary material, which is available to authorized users.

## Background

*Staphylococcus aureus* is a bacterial species often associated with primate hosts, and specific sequence types (ST) can be found frequently living on different domestic animals [1]. In humans, *S. aureus* is frequently isolated from nasal membranes and skin as residents or transients [2]. Because of its association with clinically significant infections and foodborne diseases [3, 4], *S. aureus* has been extensively investigated, leading to massive datasets on its species diversity. After the genetically divergent ST 75 was first reported in Australia in 2002 [5], many other isolates genetically related to clonal complex 75 (CC 75) were described in Belgium [6], Cambodia [7], China [8], Fiji [9, 10], France [11], French Guiana [12], New Zealand [9], Thailand [13, 14], Trinidad & Tobago [15], and the UK [16]. Genomic data tracking showed that this lineage also appeared in the United States [17]. Meanwhile, another *S. aureus* lineage has recently been recovered from nonhuman primates [18, 19] and bats [20] in Africa. Recently, these two genetically divergent lineages have received formal taxonomic classification and were recognized as *S. argenteus* and *S. schweitzeri*, respectively, two novel species within the *S. aureus* complex (SAC) [16].

*S. argenteus* cannot be distinguished from *S. aureus* using routine diagnostic microbiology identification methods [7, 8, 10, 12, 13], and PCR amplification of the gene *nucA*, which is used as a standard confirmatory marker for *S. aureus*, may be positive in *S. argenteus* [16]. However, considerable difficulties were observed in amplification of some multilocus sequence typing (MLST) gene loci from *S. argenteus* using standard MLST primers used for typing *S. aureus* [7, 21]. These difficulties may result in *S. argenteus* isolates being excluded from or misidentified as *S. aureus*. Data on clinical features of *S. argenteus* infection are limited, but these studies indicated that *S. argenteus* was associated with skin and soft tissue infections, nosocomial infections, invasive staphylococcal sepsis, and even death [13, 14, 22]. Genes encoding the Panton-Valentine leukocidin (PVL) cytotoxin and staphylococcal enterotoxin B (SEB) were detected in *S. argenteus* isolates [11, 12, 14]. Community-acquired methicillin-resistant “*S. aureus*” (CA-MRSA) isolates, which were subsequently characterized as *S. argenteus*, were predominant in remote aboriginal communities of Australia [23]. Therefore, there is no doubt that *S. argenteus* is a threat to human health.

The emergence of *S. argenteus* and *S. schweitzeri* results in the need to determine whether they should be distinguished from *S. aureus* in routine practice. Chantratita et al. suggested that this might be necessary if the infection is associated with different clinical manifestations, and/or requires different antimicrobial regimens [14]. According to this suggestion, *S. schweitzeri* is clearly separated from the other two species though one isolate was obtained from human (ST 1822, cause no infection) [24], but *S. argenteus* is difficult to make a judgment based on current available data. In this study, we sequenced the genomes of five *S. argenteus* isolates, and performed a genomic comparison among SAC, so as to clarify their evolutionary relationships, to evaluate their pathogenic potential, and to identify genomic differences. This study is expected to establish an exploratory evolutionary framework regarding the evolution of pathogenicity of SAC.

## Results

### Genomic features of SAC species

**Fig. 1:**
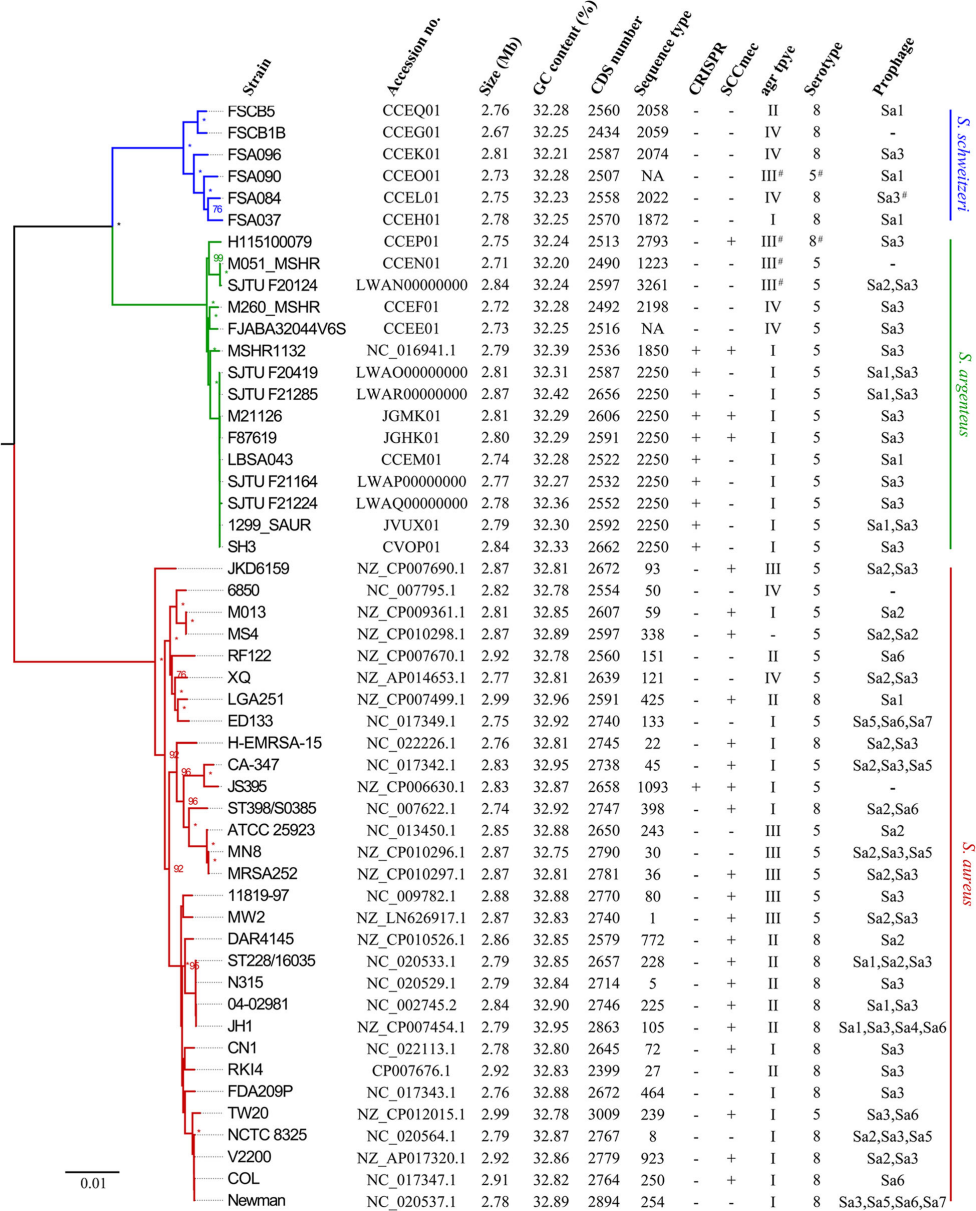
Phylogenetic relationship, genome information, and relevant typing information of SAC species. A maximum likelihood phylogenetic tree of SAC was constructed based on concatenated deduced amino acid sequences of 1375 single copy core genes of the 51 strains used in this study. Bootstrap values (expressed as percentages of 1000 replications) greater than 70% are shown at the branch points and the asterisk (*) indicates a bootstrap value of 100. The color-coded clades denote different species: *red*, *S. aureus*; *green*, *S. argenteus*; *blue*, *S. schweitzeri*. *S. simiae* was used as an outgroup (not shown). *S. argenteus* strains, SJTU F20124 (GenBank accession number: LWAN00000000), F20419 (LWAO00000000), F21164 (LWAP00000000), F21224 (LWAQ00000000), and F21285 (LWAR00000000) were sequenced as part of this study. For Sequence type, CRISPR, SCCmec, *agr* type, Serotype, and Prophage data: -, not detected/absent; +, detected/present; NA, data not available in the MLST database; and #, the type is close but divergent from the indicated subtype

The sequences of 51 SAC genomes were used in this study and shown in Fig. 1. The draft genome sequences of five *S. argenteus* strains previously identified in our laboratory [8] were sequenced in this study. Thirty *S. aureus* genomes, which represent a genetically diverse collection of strains of several different STs (Additional file 1: Figure S1), were selected from the complete genome sequences available in the NCBI genome database. Ten *S. argenteus* and six *S. schweitzeri* genomes (complete or draft) were also downloaded from NCBI.

For *S. aureus*, the genome sizes varied from 2.74 Mb (strain ST398/S0385) to 2.99 Mb (TW20), the GC contents varied from 32.75% (MN8) to 32.96% (LGA251), and the number of coding sequences (CDS) per genome varied from 2399 (RKI4) to 3009 (TW20) (Fig. 1). For *S. argenteus*, the genome sizes varied from 2.71 Mb (M051_MSHR) to 2.87 Mb (SJTU F21285), the GC contents varied from 32.20% (M051_MSHR) to 32.42% (SJTU F21285), and the number of CDSs per genome varied from 2490 (M051_MSHR) to 2662 (SH3) (Fig. 1). For *S. schweitzeri*, the genome sizes varied from 2.67 Mb (FSCB1B) to 2.81 Mb (FSA096), the GC contents varied from 32.21% (FSA096) to 32.28% (FSCB5), and the number of CDSs per genome varied from 2434 (FSCB1B) to 2587 (FSA096) (Fig. 1).

The genome size, GC content and number of CDSs were significantly different between *S. aureus* and the two new species (*p* < 0.01), while no significant differences were observed between *S. argenteus* and *S. schweitzeri* (*P* > 0.01; Additional file 2: Figure S2). The number of genomes used in this study was limited, particularly for *S. argenteus* and *S. schweitzeri*. Furthermore, some genome sizes and CDS numbers were estimated from draft genome assemblies and remained incomplete. Nevertheless, the difference in GC content, which is less affected by the quality of genome assembly, suggests an apparent divergence between *S. aureus* and the other two members of the SAC.

### Pan-genome of SAC species

To facilitate a genomic comparison and achieve a sound comparison at the whole genome scale, the pan-genome of SAC was defined. A pan-genome was previously described to include two distinct components, the core and variable genomes, which represent the essence and the diversity of the population, respectively [25]. The core genome consists of genes that are common to all strains while the variable genome is composed of genes absent or present at least in one strain, due to either gene loss or acquisition [26]. Here we expanded the classification of the SAC pan-genome into four genomic components: 1) core genes (CR), present in all strains; 2) core variable genes (CV), present in all strains with at least one pseudogene; 3) variable genes (VR), present in at least two strains and absent at least in one strain; and 4) unique genes (UQ), present in only one strain. The pan-genome of the 51 SAC genomes was grouped into 4249 homologous gene families, including 1671 CRs, 328 CVs, 1634 VRs, and 616 UQs (Additional file 3: Table S1; Additional file 4: Figure S3). The pan-genome of the 30 *S. aureus* genomes was grouped into 3966 homologous gene families, including 1752 CRs, 330 CVs, 1329 VRs, and 555 UQs. The sizes of CRs, CVs, VRs and UQ were also calculated for *S. argenteus* and *S. schweitzeri* (Additional file 5: Figure S4). The category analysis of the Cluster of Orthologous Groups (COGs) [27] for the pan-genome of the SAC and each species showed similar profiles.

Clearly, the pan-genomes for each group were all open (i.e., not complete), due to the limited number of genomes included in the analyses. The accumulation curves and pan-genome sizes largely depend on sampling size and diversity [25]. In this study, *S. aureus* was better sampled than *S. argenteus* and *S. schweitzeri* in both the number of strains and strain diversity. For example, of the fifteen *S. argenteus* genomes examined, nine were ST2250 demonstrating little genome diversity (see below). Therefore, a brief comparison of the pan-genome is shown in Additional files 6: Figure S5, and the details will not be discussed here. However, it is worth noting that the 1999 common gene families (CR plus CV) in the SAC pan-genome were approximately three quarters of the size of an individual SAC genome, and the 2578 sharing gene families (genes present in at least one genome of each species, including CR, CV and partial VR) were approximately equivalent to the CDS number of an individual SAC species genome. This is indicative of high level of sharing of homologous gene families between the species and strains of the SAC. It is predictable that *S. aureus*, *S. argenteus* and *S. schweitzeri* share many biological characteristics, many of which have been demonstrated in previous studies as a common phenotypes [16].

### Core genome of the SAC species

**Fig. 2:**
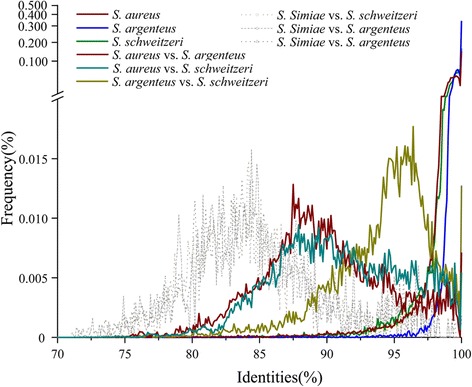
Pairwise bANI of SAC species. Pairwise bANI of *S. simiae, S. aureus*, *S. argenteus* and *S. schweitzeri* were determined based on the 1375 common single copy genes. For comparison of *S. simiae to S. aureus*, *S. argenteus* and *S. schweitzeri*, the mean bANI values were 84.63 ± 5.18% (median 84.14%), 84.89 ± 5.25% (84.26%), and 84.85 ± 5.19% (84.17%), respectively. Among the members of the SAC 1596 common genes were used determine the bANI. Interspecific mean bANI values were as follows: *S. aureus*, 98.76 ± 1.92% (median 99.23%); *S. argenteus*, 99.6 ± 0.85% (99.79%); and *S. schweitzeri*, 98.87 ± 1.74% (99.38%). The intraspecific mean bANI values were as follows: *S. aureus* vs. *S. argenteus*, 89.79 ± 4.63% (median 89.39%); *S. aureus* vs. *S. schweitzeri*, 90.92 ± 4.77% (90.64%); and *S. argenteus* vs. *S. schweitzeri*, 94.08 ± 3.69% (94.81%). The group interval was 0.1%

Similar metabolic pathways and phenotypes between species may be the result of frequent genetic exchanges, such as horizontal gene transfer (HGT) mediated by bacteriophage [28, 29], or limited evolutionary time for divergence from a common ancestor. HGT mediated by mobilome mainly contributes to the VRs and UQs, respectively as follows (Additional file 4: Figure S3; Additional file 5: Figure S4, code X): SAC, VRs and UQs accounting for 62.7% and 34.7% of the genes assigned to X class, respectively; *S. aureus*, 63.5% and 33.6%; *S. argenteus*, 61.5% and 32.3%; and *S. schweitzeri*, 64.8% and 27.8%. Also, it is believed that distinct species are separated by apparent genetic distances that can act as barriers to recombination [30–32]. To better understand the genetic distances, the average nucleotide identities based on BLAST (bANI) were calculated using the core genes shared among SAC species (Fig. 2). Most of the intraspecific sequence identity values were greater than 95%, which was consistent with the species definition of an ANI cutoff of 95–96% [33]. The interspecific values from different groups formed three peaks similar to normal distributions and exhibited apparently differences: the peak of *S. argenteus* versus *S. schweitzeri* was near 95% while those of *S. argenteus* versus *S. aureus* and *S. schweitzeri* versus *S. aureus* were 86–89%. The core genome of *S. schweitzeri* (90.92 ± 4.77%) was closer related to that of *S. aureus* than *S. argenteus* (89.79 ± 4.63%, Fig. 2). Notably, in pairwise comparisons between species, bANI values greater than 95% were a small but notable part of the total. To determine whether these identity values (> 95%) were generated from highly conserved house-keeping genes (evolving very slowly), the pairwise distance among alleles of each gene family was tested. It was found that the number of gene families with an average interspecific identity greater than 95% was 188 between *S. aureus* and *S. argenteus*, 307 between *S. aureus* and *S. schweitzeri*, and 698 between *S. argenteus* and *S. schweitzeri* (161 in common). Meanwhile, 34 gene families between *S. aureus* and *S. argenteus*, 33 between *S. aureus* and *S. schweitzeri*, and 80 between *S. argenteus* and *S. schweitzeri* (28 in common) had the same amino acid sequence. It indicated that the bANI values greater than 95% were mainly due to slowly evolving genes, but horizontal gene transfer between species may also be present.

The species of SAC have identical 16S rRNA gene while MLST and other house-keeping genes can be used to distinguish them from each other [16, 21]. Nevertheless, the MLST loci are just a small part of the genome. Here, an upgraded MLST method [34] was used to compare 1375 single copy core genes of SAC and *S. simiae* (used as an outgroup), which was previously considered as the closest taxa to *S. aureus* [35], were used to infer phylogeny. The Maximum Likelihood (ML) algorithm was used and a well-supported topology was obtained (Fig. 1). *S. argenteus* and *S. schweitzeri* clustered together (3.05% in distance based on amino acid sequence) and then clustered with *S. aureus* (4.42% in distance) in the core-genome tree, with perfect supporting bootstrap values (100%). *S. simiae* grouped far away from SAC with 21.34% in distance. This suggests that SAC members separated from each other much later than from other staphylococci, resulting in species divergence from a shared global core genome of a common ancestor.

### Population structure

**Fig. 3:**
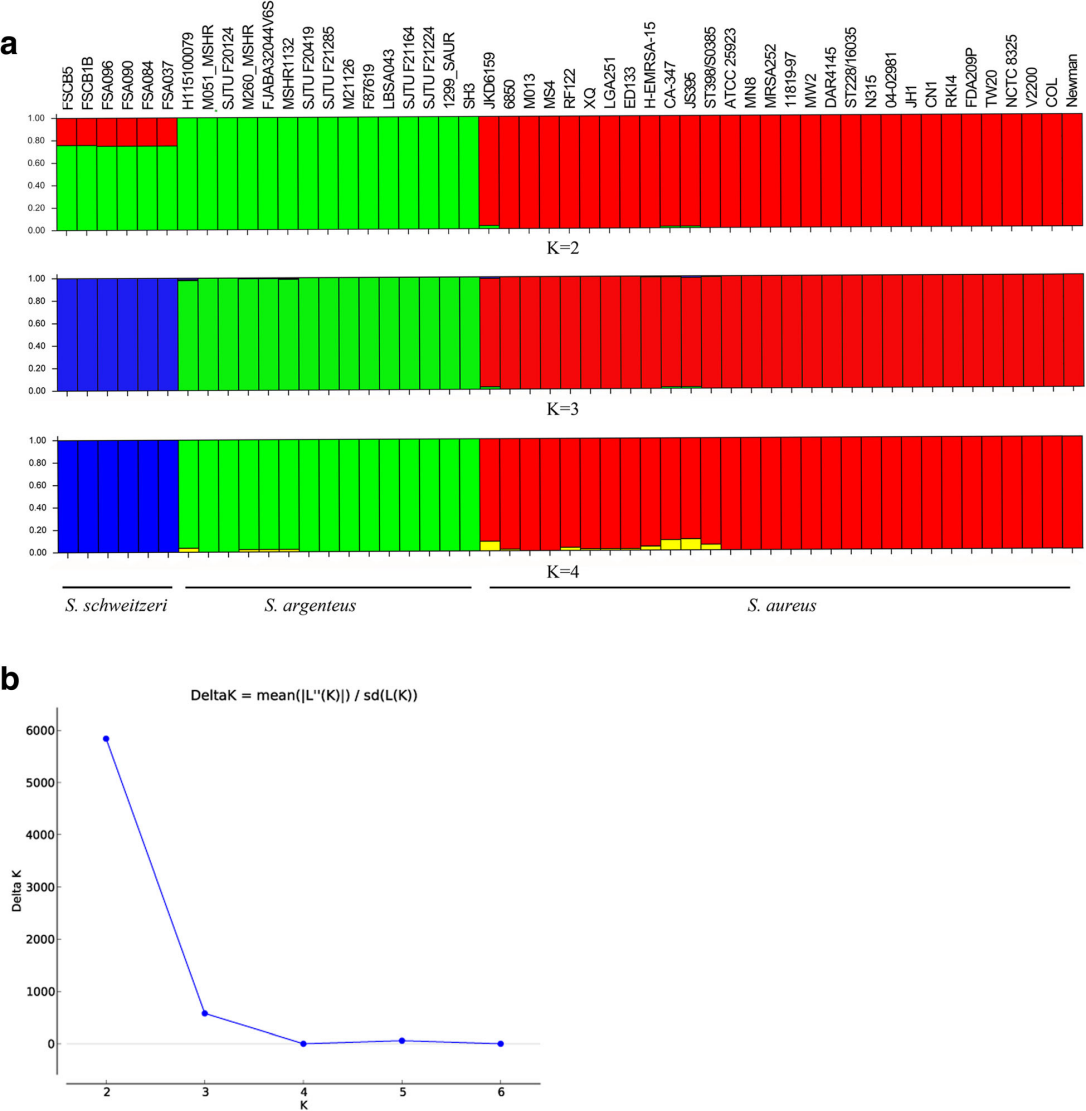
Population structure of *Staphylococcus aureus* complex. **a** The population memberships of the inspected species for a priori defined number of clusters K = 2–4 inferred by the Structure software. Each individual is represented by a thin vertical line divided into K colored segments that represent the individual’s estimated membership fractions in K clusters. Populations are labeled below the figure. **b** The detection of the true number of clusters inferred by the Structure software and set ΔK = *mean*(|*L"*(*K*)|)/ *sd*(*L*(*K*)) as a function of K. ΔK attains its highest value when K = 2

The program Structure version 2.3.4 [36], which implements a model-based clustering method, was used to infer the SAC population structure using genotype data of the 1596 single copy core genes. Structure Harvester was used for collating results generated by the program Structure [37]. The ΔK values, an ad hoc quantity related to the second order rate of change of the log probability of data with respect to the number of clusters, were calculated by Structure Harvester to detecting the number of K populations that best fit the data. A higher ΔK means a better fitness of the K value. The highest ΔK value (= 5840) emerged when K = 2 (Fig. 3). It indicated that staphylococcal strains investigated here fall into two distinct populations corresponding to *S. aureus* and *S. argenteus*, respectively (Fig. 3a). Six individuals of *S. schweitzeri* seemed to be hybrids between *S. aureus* (24–25%) and *S. argenteus* (75–76%). Five *S. aureus* strains, JKD6159, CA-347, H-EMRSA-15, JS395 and ST398/S0385, appeared to be hybrids among the *S. aureus* (red) and the *S. argenteus* (green) populations, with all individuals showing < 3.2% ancestry from *S. argenteus* (green) population. None of *S. argenteus* strains was found to be a hybrid. We also observed a higher ΔK value (= 579) when K = 3, and the three distinct populations corresponded to *S. aureus*, *S. argenteus* and *S. schweitzeri*, respectively (Fig. 3a). None of *S. schweitzeri* strains was found to be a hybrid, but four strains of *S. argenteus* were hybrids showing < 1.5% ancestry from *S. aureus* (red) and *S. schweitzeri* (blue) populations. And five strains of *S. aureus* were hybrids showing < 2.3% ancestry from *S. argenteus* (green) and *S. schweitzeri* (blue) populations. These results suggested that *S. argenteus* had an independent population structure with rare recombination occurred between the core genomes of *S. argenteus* and the other two species during the speciation. Notably, *S. schweitzeri* was found to be hybrids when K = 2, and it was independent population when K = 3, which implied this species might be the descendant of an ancient hybrid between *S. aureus* and *S. argenteus*. Nevertheless, it was not further discussed in this study, considering limited genomes and knowledge on this species.

### SAC species-specific genes

The species-specific genes (present or absent in all strains of one species) among SAC species were identified and shown in Table 1. We arbitrarily defined one gene is acquired in one species if the gene (including related pseudogene) is not found in the other species, and one gene is lost in one species if the gene is found in the other species. It shoud be a gene loss event if a pseudogene is found in one species and the related gene is present in the other species. Based on this definition, *S. aureus* acquired 24 unique genes while *S. argenteus* and *S. schweitzeri* acquired six and zero, respectively. *S. aureus* lost four genes while *S. argenteus* and *S. schweitzeri* lost 16 and ten, respectively. Some newly acquired genes were not required for growth under some conditions and became pseudogenes in some strains, for example, a gene encoding a protein N-acetyltransferase was acquired by but became pseudogenes in some strains (gene family 2832 in Table 1). Meanwhile, some genes were inherited from the common ancestor but were kept as pseudogenes in all strains of one species (Table 1). These pairwise species-specific genes and single species-specific genes were reannotated in COG, UniPro database, and KEGG Automatic Annotation Server [27, 38, 39], but most of their functions were unknown, especially those of *S. aureus* (Table 1). *S. aureus* and *S. schweitzeri* are different from *S. argenteus* in that *S. argenteus* lacks the *crtOPQMN* operon encoding the staphyloxanthin pathway (resulting in yellow colonies) [8, 16, 22]. Our data also indicated that white-colony forming *S. aureus* isolates might have resulted from inactivation of *crtM*, *crtN*, and *crtO* (Table 1) or a non-functional *sigB* operon [40]. Some species-specific genes are also prevalent in coagulase-negative staphylococci (CoNS) with low identities (< 90%), indicating that they might be inherited from the common staphylococcal ancestor, with some species of SAC losing them during speciation. The other species-specific genes, which were absent or present in a limited number of CoNS, probably were gained by HGT. Interestingly, all *S. aureus*-specific genes, most encoding small proteins (< 80 aa) with unknown functions, were absent in other staphylococci.

**Table 1:** Species-specific gene list of *S. aureus* complex (SAC)

Representitive sequence	Function	*S. aureus* ^a^	*S. argenteus* ^a^	*S. schweitzeri* ^a^	Prevelence in CoNS^b^	Gene family^c^
SAOUHSC_01325	Unknown	+	-#	-#	–	2409
SAOUHSC_01339	Unknown	+	–	–	–	2410
SAOUHSC_01834	Unknown	+	–	–	–	2411
SAOUHSC_01853	Unknown	+	–	–	–	2412
SAOUHSC_01883	Unknown	+	-#	-#	–	2413
SAOUHSC_02518	Unknown	+	–	–	–	2415
SAOUHSC_02657	Unknown	+	–	–	–	2416
SAOUHSC_02734	Unknown	+	–	–	–	2417
SAOUHSC_02890	Unknown	+	–	–	–	2418
SAOUHSC_02934	Unknown	+	–	–	–	2419
SAOUHSC_00208	Unknown	+	-#	–	–	2427
KQ76_RS08635	Unknown	+	-#	-#	–	2439
SAOUHSC_02533	Unknown	+	–	–	–	2442
KQ76_RS11720	Unknown	+	–	–	–	2443
KQ76_RS13490	Unknown	+	–	–	–	2446
KQ76_RS13520	Unknown	+	–	–	–	2447
SAOUHSC_02332	Unknown	+	-#	-#	–	2504
SAOUHSC_01003	Unknown	+*	–	–	–	2433
SAOUHSC_01357	Unknown	+*	–	–	–	2436
SAOUHSC_02705	Unknown	+*	–	–	–	2444
SAOUHSC_00238	Unknown	+*	–	–	–	2460
SAOUHSC_01603	Unknown	+*	–	–	–	2465
SAOUHSC_01770	Unknown	+*	–	–	–	2467
SAOUHSC_A02189	Unknown	+*	-#	-#	–	2472
SAOUHSC_01765	Unknown	+*	–	–	–	2495
KQ76_RS11950	Unknown	+*	–	–	–	2499
SAOUHSC_02572	Unknown	+*	–	–	–	2518
SACOL_RS11100	Unknown	+*	–	–	–	2779
KQ76_RS10810	Unknown	+*	–	–	–	3372
SAOUHSC_A02577	Unknown	+*	–	–	–	2603
SAMSHR1132_RS10135	Unknown	–	+	–	–	2769
SAMSHR1132_RS12985	NADP-dependent 3-hydroxy acid dehydrogenase	–	+	–	*S. carnosus*, *S. condimenti* (2)	2770
SAMSHR1132_RS12990	Fermentation-respiration switch protein FrsA	–	+	–	*S. simiae*, *S. intermmedius* (5)	2771
SAMSHR1132_RS13595	Unknown	–	+	–	*S. delphini*	2775
SAMSHR1132_RS03050	Uncharacterized protein YqcI	–	+*	–	*S. warneri*, *S. epidermidis* (2)	2814
SAMSHR1132_RS02070	Protein N-acetyltransferase, RimJ/RimL family	–	+*	–	*S. capitis*, *S. warneri* (13)	2832
SAMSHR1132_RS13575	Carboxylesterase type B	–	+	+	–	2599
SAMSHR1132_RS13600	Unknown	–	+	+	–	2600
SAMSHR1132_RS04625	Unknown	–	+	+	*S. simiae*	2774
ERS140266_RS13125	Unknown	–	+*	+	*S. lugdunensis*, *S. intermedius* (6)	2693
SAOUHSC_00229	Iron-sulfur cluster repair protein ScdA	+	–	+	*S. simiae*, *S. epidermidis* (22)	2276
SAOUHSC_00237	Methyltransferase domain protein UbiE	+	–	+	*S. simiae*, *S. agnetis* (11)	2277
SAOUHSC_00492	Unknown	+	–	+	–	2313
SAOUHSC_02880	4,4′-diaponeurosporenoate glycosyltransferase CrtQ	+	–	+	*S. simiae*, *S. caprae* (6)	2280
SAOUHSC_02881	Diapolycopene oxygenase CrtP	+	–	+	*S. simiae*, *S. capitis* (16)	2281
ERS140266_RS00255	Dehydrosqualene desaturase CrtN	+*	–	+	*S. simiae*, *S. lugdunensis* (17)	2294
ERS140266_RS00260	Dehydrosqualene synthase CrtM	+*	–	+	*S. simiae*, *S. capitis* (8)	2295
ERS140266_RS00275	Glycosyl-4,4′-diaponeurosporenoate acyltransferase CrtO	+*	–	+	–	2296
ERS140266_RS02765	Unknown	+*	–	+*	–	2488
ERS140266_RS07180	Unknown	+*	-#	+	–	3141
SAOUHSC_00355	Uncharacterized protein YxeA	+	+	–	–	2176
SAOUHSC_00867	Unknown	+	+	-#	–	2198
SAMSHR1132_RS12095	Probable amino acid-proton symporter YbeC	+*	+	–	*S.simiae*, *S. caprae* (20)	2192
SAMSHR1132_RS03980	Uncharacterized protein YwqG	+*	+	–	*S. lugdunensis*	2211
SAMSHR1132_RS02265	Unknown	+*	+	-#	–	2234

^a,^*,present as pseudogene in some strains; #, gene function lost, pseudogenes in all strains. ^b^CoNS, coagulase negative staphylococci; number in parentheses indicates how many strains of the non-SAC Staphylococcus species harbor a homolog with both identity and percentage of matches > 50%. ^c^gene family numbers come from the complete detailed gene list presented in Additional file 3: Table S1

### Mobile genetic elements (MGE), *agr* and the capsular polysaccharide gene cluster

Temperate bacteriophages of the major *Siphoviridae* family play an important role in the pathogenicity of *S. aureus* by mediating the HGT of virulence factors [29]. The detection of genes encoding these prophages’ integrases, integrase groups Sa1–3, indicated the presence of the prophages in the genomes of *S. argenteus* and *S. schweitzeri* (Fig. 1), which take many virulence genes along with them [29]. Methicillin-resistant *S. aureus* (MRSA) strains are of particular importance because they are a leading cause of nosocomial infections worldwide [41]. Methicillin resistance in MRSA is due to an acquisition of the staphylococcal cassette chromosome *mec* (SCC*mec*) element. Evidence for the presence of SCC*mec* in *S. argenteus* has been reported previously [6, 23] and is further substantiated in this study (Additional file 3: Table S1; *mecA*, gene family 2509; *ccrA*, family 2525; *ccrB*/*C*, family 2583). The clustered regularly interspaced short palindromic repeats-CRISPR-associated proteins (CRISPR-*Cas*) modules are adaptive immunity systems that are present in many archaea and bacteria [42], but are not very common in *S. aureus* (Fig. 1). Some strains of *S. argenteus* and *S. aureus* harbor a characteristic *cas10/csm1* (gene family 2896 in Additional file 3: Table S1), and a subtype of CRISPR-*Cas* system III was present in them according to previous classification [42], especially strains of ST2250. Related genes of *S. argenteus* CRISPR-*Cas* system are a somewhat divergent from those of *S. aureus* strain JS395 (nucleotide sequence similarity 93.8%). However, further comparison with other *S. aureus* strains in NCBI showed that some *S. aureus* strains, such as M1169 (JEKD00000000) possessed a CRISPR-*cas* system with high similarity to that of *S. argenteus* (> 99%), suggesting that CRISPR loci can be mobilized and can transfer between different but closely related species.

**Fig. 4:**
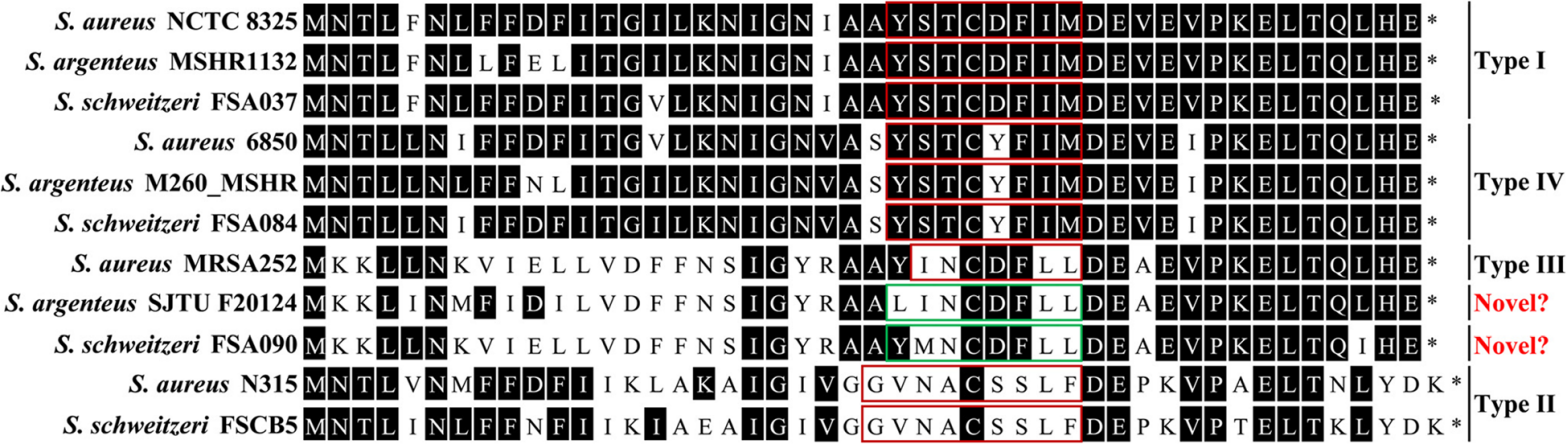
Comparisons of the predicted AgrD amino acid sequences from SAC species and *agr* types. Strains of the same *agr* type for each species have a same common sequence and are represented by a single sequence in this figure. Known autoinducing peptide (AIP) molecule sequences are marked in *red rectangles*. Potential novel AIP molecule sequences are marked in *green rectangles*

The staphylococcal *agr* locus encodes a quorum sensing system (QS) that controls the expression of virulence and other accessory genes by a classical two-component signaling module, and it is distinct among staphylococcal species [43]. However, *S. argenteus* harbors *S. aureus arg* type I and IV while *S. schweitzeri* type I, II, and IV (Fig. 1). Further analysis suggested that *agrA* was highly conserved within species and had no common sequence differences among *agr* types. The *agrB* gene may suffer ancient HGT among species, especially that of *S. schweitzeri agr*I and ‘*agr*III’ (maybe novel type), while the *agrCD* genes cluster primarily by *arg* type and secondarily by species (Additional file 7: Figure S6). The *agrD* alignment is shown in Fig. 4. It looks like that the evolution of agr locus among SAC species was polyphyletic. Based on this conjecture, the same *agr* types in different SAC species would have evolved independently. Gene *agrD* produces a ribosomal propeptide of which the middle section encodes the seven to nine residue autoinducing peptide (AIP) used as a QS signal molecule [43]. We found two possible novel types of *agr* most closely related to type III *agr* (Fig. 4). These novel *agr* types were present in three strains of *S. argenteus* (SJTU F20124, H115100079 and M051_MSHR) and one strain of *S. schweitzeri* (FSA090) (Fig. 1).

Like many other bacterial pathogens, *S. aureus* produces capsular polysaccharide (CP) that enhances its resistance to clearance by host innate immune defenses, with strains of serotypes 5 and 8 being the most among clinical isolates [44]. We compared the CP gene clusters among SAC members (Additional file 8: Figure S7). For most of the genes common region of the CP gene cluster (*capA-P*, see reference 4 for a review), *S. aureus*, *S. argenteus* and *S. schweitzeri* were all phylogenetically distinct from each other. The exceptions were for the *capM* and *capN* genes where the phylogenetic trees showed that *S. aureus* and *S. schweitzeri* clustered together and HGT is likely to have occurred. In the serotype specific gene region (*capHIJK*), *S. aureus* was clearly divided into two groups consisting of strains of serotype 5 and 8, respectively. Strain *S. schweitzeri* FSA090 clustered with 14 *S. argenteus* strains in a group more closely related to *S. aureus* strains of serotype 5. Similarly, one strain *S. argenteus*, H115100079, clustered with five *S. schweitzeri* strains in a group most closely related to *S. aureus* strains of serotype 8. These results suggest that *S. argenteus* and *S. schweitzeri* may express their own serotype(s) that could have been derived from the ancestors of *S. aureus* serotype 5 and 8, respectively.

### Virulence gene distribution

**Fig. 5:**
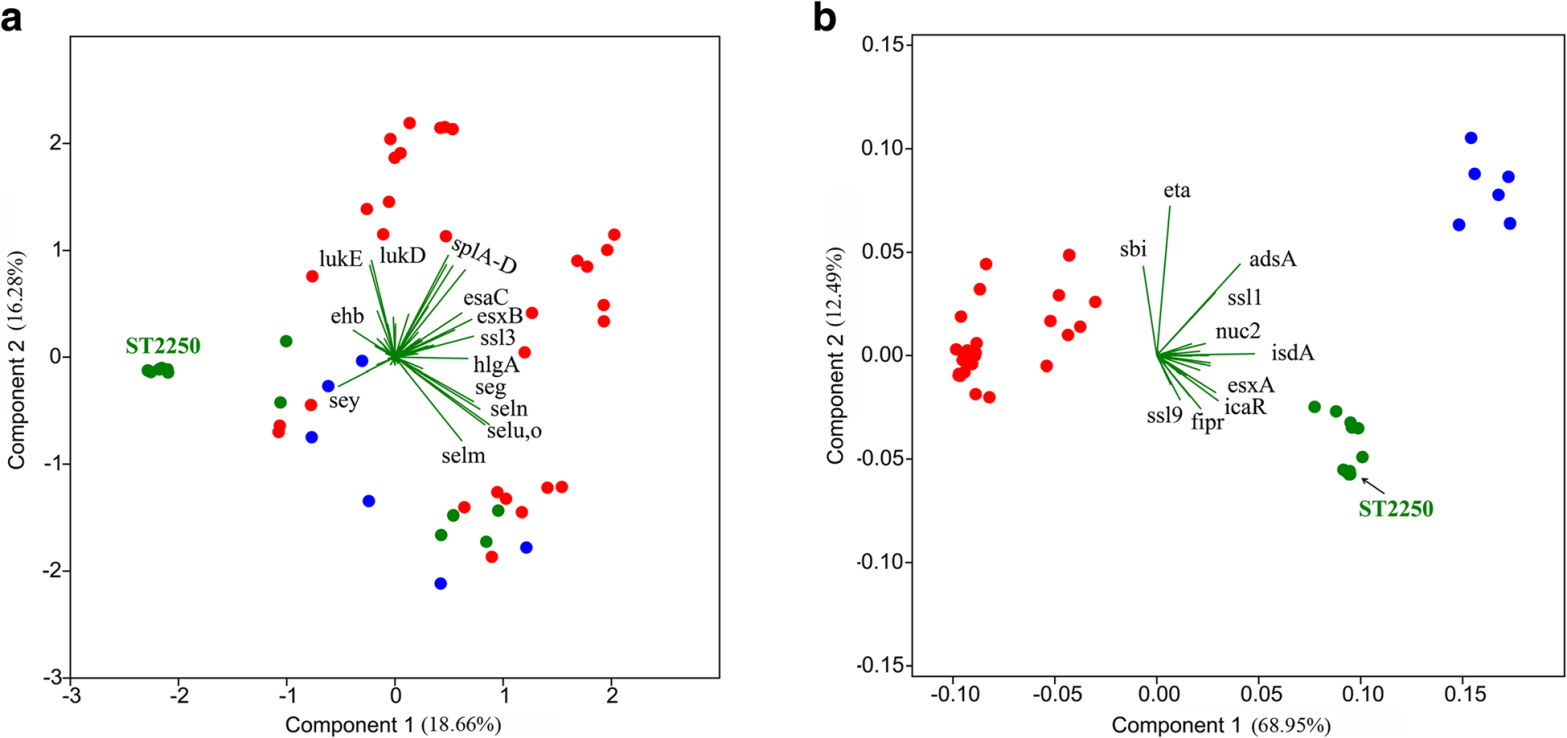
Principal Component Analysis of SAC virulence genes. **a** PCA based on the presence (1) and absence (0) of 106 virulence genes; **b** PCA based on the mean distance of one strain against the rest 50 strains among 30 core virulence genes. Five genes, *sed*, *see*, *sej*, *selr* and *etb*, were excluded from the gene because they were not present in any strain. Species are marked using different colored dots: *S. aureus* (*red*), *S. argenteus* (*green*) and *S. schweitzeri* (*blue*). The cluster of dots representing *S. argenteus* ST2250 strains is indicated by the *arrow*. The genes that provide the best strain differentiation are shown in the biplot

A hallmark of *S. aureus* infections is their frequent recurrence, which results from the manipulation of host immune responses by dozens of virulence genes [45]. Several virulence genes have been reported in *S. argenteus* and *S. schweizeri*, such as *nuc*_*m*_, *pvl*, *sak*, *scn*, *seb*, *sec*, *se*l*k*, *se*l*q*, and *tsst* [6, 11, 14, 18, 46], but the presence of many other potential virulence genes have not been tested for yet. Here, we performed a systematic investigation of genome sequences for the virulence gene distribution among SAC. The results indicated that (Additional file 9: Table S2): of 111 virulence genes previously reported in *S. aureus*, only five were not found in the genome sequences of any strain examined in this study, 92 (82.9%) were present in at least two SAC species, 85 (76.6%) in *S. argenteus*, and 86 (77.5%) in *S. schweizeri*. Of the 19 (17.1%) genes absent in *S. argenteus* or *S. schweizeri*, 11 encode enterotoxins or related superantigen-like proteins, and four encode leukocidins related proteins. Besides, of the 71 (64.0%) genes that were present in at least two species and at least three strains per species, 56 (50.5%) showed a significant difference in nucleotide sequence identity in a pairwise comparison of genes between species (*p* < 0.01). It is notable that nine genes (*clfA*-*B*, *fnbA*-*B*, *sdrC*-*E*, *spa* and *coa*) have repeat regions of uneven numbers in some strains, of which a subtyping method targeting *spa* gene (coding *Staphylococcus* Protein A) is widely used to characterize *S. aureus* isolates [47]. This could result in inaccurate identification of the genetic divergence based on these genes because of difficulties to align homologous nucleotide loci and a rapid change in the number of repeats within these regions. The principal component analysis (PCA) was employed to evaluate the differences in gene content and overall divergence of virulence genes among SAC members. The results suggested that the virulence gene content of each SAC species did not differ from each other while the divergence in core virulence gene is noteworthy (Fig. 5). In fact, most virulence genes were divergent at nucleotide sequence level among *S. aureus*, *S. argenteus* and *S. schweitzeri* (please find the ML trees in Additional file 9: Table S2). However, HGT may occur in some non-MGE-associated virulence genes, such as *nucA* of *S. aureus* JS395.

Overall, the *S. argenteus* and *S. schweitzeri* pan-genome harbor all the virulence genes expressing the essential functions required for the pathogenicity in *S. aureus*. For examples, *icaA-D* encodes polysaccharide biosynthesis, which is critical to biofilm elaboration [48]; *esaA-C*, *essA-C*, and *esxAB* encodes the ESAT-6 system for secretion of exoenzymes or exotoxins into the extracellular milieu [49]; *isdA-G* and *srtB* code for heme uptake [50]. The genomic islands *ν*Saα and *ν*Saβ are also present in these two species, which carry many virulence determine genes [4]. Most of the virulence genes absent in the *S. argenteus* and *S. schweitzeri* pan-genome code for enterotoxins and other exotoxins (Additional file 9: Table S2), which are usually located in MGE and easily acquired or lost.

### Geographic characteristics of *S. argenteus* ST2250

**Fig. 6:**
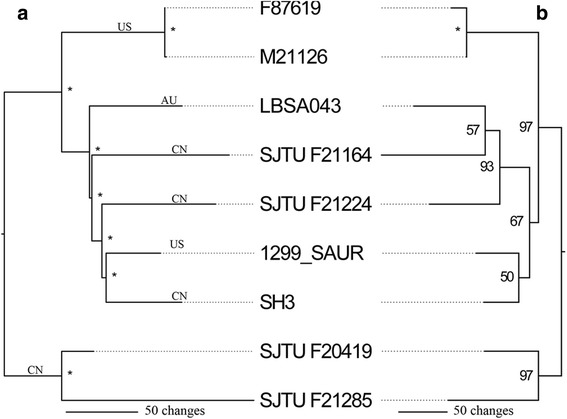
Maximum likelihood phylogenies of nine *S. argenteus* ST2250 genomes. Phylogenies were inferred from (**a**) the concatenated SNP-containing genes of core genome and (**b**) a presence/absence of gene content matrix. Numbers near each node correspond to bootstrap values in percentage of 1000 replications and the asterisk (*) indicates a bootstrap value of 100%. Geographical origins of the strains are marked on the branches: US, United States; AU, Australia; CN, China

*S. argenteus* ST2250 was the most frequent lineage isolated, and strains of this ST have a broad geographic distribution [6, 8, 11, 13–17]. The widespread distribution of *S. argenteus* ST2250 may have occurred long ago or may have occurred only recently. Signatures from core housekeeping genes and variable genes suggest that geographic barriers promote divergence among microbial populations in the environment [26, 51]. However, increased mobility of human populations may break these geographical barriers for pathogenic bacteria [52]. To clarify the question as to why *S. argenteus* ST2250 was well distributed, the core and variable genes were further analyzed. The core genome of ST2250 contained 2348 single copy gene families, of which the concatenated alignment had a length of 2.07 M bp and 1177 single nucleotide polymorphisms (SNP). However, more than a half of the SNPs (653 bp) were due to indel variation located in the tandem repeat regions of three genes, including a hypothetic serine protease gene (gene family 1742 in Additional file 3: Table S1), a serine-rich adhesin for platelets gene (*sraP*, gene family 2098), and a hypothetic membrane anchored protein gene (gene family 2132). Due to the high rate of mutation, these loci were excluded from the analysis. The remaining 524 SNPs were used to infer maximum likelihood phylogenies (Fig. 6a). This phylogenetic tree matches the core gene nucleotide phylogeny (Fig. 1) very well with significant bootstrap support, but does not resolve a distinct population in each geographical region. Strains SH3, SJTU F21164 and F21224 were isolated from China (CN) and clustered with LBSA043 from Australia (AU) and 1299_SAUR from America (US). The presence/absence of gene content matrix based on variable genes also showed similar clustering (Fig. 6b). Notably, the number of SNPs was very small compared with *S. aureus* ST239 lineage, which were also distributed intercontinentally and from which 4310 SNPs were found among the isolates from different countries [52]. By comparison, only 382 VR and UQ gene families were identified in the *S. argenteus* pan-genome, of which strain LSBA043 was present in the least gene families (43) and strain SH3 in the most (167). All 382 gene families were annotated by ACLAME web site [53] and homologues of 247 were reported to be located in MGE. These results demonstrated that isolates of *S. argenteus* ST2250 lineage were very closely related and might have spread internationally by human hosts.

## Discussion

Isolates of *S. argenteus* and *S. schweitzeri* were originally classified as parts of lineages of *S. aureus* because they share many phenotype and genotype properties [8, 16]. However, when using some molecular subtyping methods, considerable difficulties were observed, such as PCR amplification of *aroE* and *glpf* in MLST [8], and *nucA* identification of *S. schweitzeri* [16]. Few large-scale virulence gene investigations on *S. argenteus* and *S. schweitzeri* have been reported, and studies relying on PCR amplification of virulence targets should be regarded with skepticism because many *S. argenteus* and *S. schweitzeri* virulence genes are significantly divergent from those in *S. aureus* (Additional file 9: Table S2). Nonetheless, there is a great deal of genetic relatedness among SAC species, and homologous genes have been leveraged to develop many broadly recognized descriptive indexes among SAC, such as MLST, SCC*mec*, *spa*, *agr*, and prophage typing. Obviously, *S. aureus*, *S. argenteus* and *S. schweitzeri* are more closely related to each other than they are to other *Staphylococcus* species, of which these descriptive indexes are very distinct, for example, *agr* and prophage types of SAC never reported to be present in other staphylococci [29, 43]. Therefore, as previously suggested [16], the term SAC can be used to describe the closely related group of species including *S. aureus, S. argenteus* and *S. schweitzeri*.

Taking SAC as a starting point, many descriptions should be re-examined, for example, MRSA could refer to any SAC isolate harboring a SCC*mec* element. There is also a need to unify other descriptive indexes as well as diagnostic and typing methods, especially virulence gene detection and molecular subtyping. For example, we recently reported redesigned *aroE* and *glpf* primers for MLST of SAC and proposed a simple PCR method for identification and differentiation of SAC isolates [8]. The gene family list generated in the current study will also accelerate the development of uniform methods for the detection of many virulence genes (Additional file 3: Table S1; Additional file 9: Table S2). However, some virulence genes (especially core genes) may evolve quickly, with several very divergent subtypes occurring among or within SAC species. For example, alleles of *S. schweitzeri nuc2* formed two distant clades, one closely related to *S. aureus* and the other closely to *S. argenteus*. Furthermore, divergent subtypes appear in many virulence genes of *S. aureus* and *S. schweitzeri* (note the high intraspecific average distance in Additional file 9: Table S2). The *nuc*_*M*_ recently identified in *S. schweitzeri* [46] is a divergent homolog of the *S. aureus nucA*, and *S. argenteus* harbors an additional *nucA* variant. Thus, the current nomenclature of *nuc*_*M*_ does not properly reflect its relationships to *nucA* (ortholog) and *nuc2* (paralog). Therefore, much effort needs to be devoted to properly name genes among species of SAC.

We demonstrated here that, *S. schweitzeri* and *S. argenteus* have a similar distance to *S. aureus*, and that they are much closer to each other than to *S. aureus* (Fig. 2; ML trees in Additional file 9: Table S2). The relationships of these two species to *S. aureus* were reflected by whole genome ANI and DNA–DNA hybridization analysis in previous study [16]. Therefore, the most recent common ancestor of all three was earlier than that of *S. schweitzeri* and *S. argenteus*. However, this relationship is in contrast to host association for SAC infection. That is, *S. aureus* and *S. argenteus* are commonly associated with human disease, while *S. schweitzeri* rarely. *S. argenteus* and *S. schweitzeri* have lower GC content compared to *S. aureus*, which may be the result of mutation and selection involving multiple factors, such as the environment, symbiotic lifestyle and aerobiosis [54]. Additionally, *S. argenteus* shows a low occurrence in many regions where it has been reported [6, 8, 13]. It seems unlikely that *S. argenteus* emerged as a human pathogen and can escape clinical monitoring until the last decade. It is more likely that *S. argenteus* may have originally been a different ecotype from *S. aureus* and a host adaption occurred to allow it to infect humans, which is also implied by a very recent report [55]. Nonethelessly, we can not deny another possibility that *S. argenteus* have been previously mistaken as *S. aureus* and failed to spread and draw enough attention.

Although MRSA of *S. argenteus* was predominant in community-acquired isolates in some regions [23], *S. argenteus* showed resistances to fewer antimicrobials than is typical in *S. aureus* [12, 14]. Thus it seems that there is currently no need to modify therapeutic regimes for *S. aureus* and *S. argenteus* infections. Nevertheless, genes responsible for antimicrobial resistance are often located in MGEs and easily acquired, enhancement of *S. argenteus* antimicrobial resistance may be just a matter of time and the rapid international spread of *S. aureus* [52], and *S. argenteus* (this study) may aggravate the situation. In this study, it was suggested that *S. argenteus* harbors all the core virulence genes of *S. aureus* (Additional file 9: Table S2), but the expression and regulations of most of these virulence factors have not yet been characterized. In addition, many species-specific genes were recognized that presently have unknown functions (Table 1). The population structure analysis (Fig. 3) suggested distinct evolution background of the core genome between *S. aureus* and *S. argenteus*. These differences may result in different mechanisms of virulence to *S. argenteus* and *S. aureus* and different invasiveness between *S. schweitzeri* and *S. aureus*, which will impact the development of approaches to targeted drug design and therapeutic regimens in the post-antibiotic era [56]. For example, it was recently shown that diapophytoene desaturase (CrtN, the second enzyme of the staphyloxanthin biosynthesis pathway) is a potential target for drug development against *S. aureus* infections [57], but, to date, this drug targets are absent in all *S. argenteus* isolates. The future development of targeted drug therapies and diagnostic tests that distinguish these two species should allow for improved patient outcomes.

*S. argenteus* was found to possess most of the virulence genes of *S. aureus* (Fig. 5; Additional file 9: Table S2), which implied a pathogenic potential similar to *S. aureus* at a genomic level, and the international spread of *S. argenteus* ST2250, most likely anthropogenically, may worsen the situation. Furthermore, species-specific genes among SAC members recognized in this study may be responsible for the different ecotypes. Further investigation on the function of these unique gene products will help determine their contribution to speciation and ecotype. It is fascinating that the diversity of the *agr* locus indicates a polyphyletic relationship among SAC, suggesting that each species of SAC was derived from more than one common ancestor [58]. However, the number of available genome sequences for *S. argenteus* and *S. schweitzeri* are still very limited and their diversity is unclear, so it was not discussed in this study.

## Conclusions

We proposed to use the term SAC to cover *S. aureus*, *S. argenteus* and *S. schweitzeri* to indicate their close relationships. Considering difficulties in subtyping and virulence gene detecting using the methods designed for *S. aureus*, much effort needs to be devoted to developing universal and robust methods among SAC species. It is found in this study that *S. argenteus* harbored most virulence genes of *S. aureus* and had spread internationally, which suggested that *S. argenteus* may have a similar pathogenic potential as *S. aureus*. However, genomic divergence was also observed, especially regions of virulence genes, which draw necessary to distinguish *S. argenteus* from *S. aureus* in routine practice. Data from this study and previous ones draws a hypothesis that *S. argenteus* should have originally been a different ecotype from *S. aureus* and a host adaption occurred to allow it to infect humans. Finally, the clinical importance of *S. argenteus* underscores the need for broader genomic epidemiological investigations. Such studies would be expected to provide clarification on the origin of *S. argenteus* and the evolution of its infectivity and pathogenicity. Additional microbiological studies are also needed to determine the species environmental niche and further elaborate mechanisms of pathogenicity.

## Methods

### Strains and genomes

The five *S. argenteus* strains were isolated from China and characterized previously [8]. The genomes were sequenced using a MiSeq 300PE sequencer (Illumina, San Diego, CA, USA) at GENEWIZ (Suzhou, China). Velvet version 1.2.10 [59], SSPACE version 3.0 [60], and GapFiller version 1–10 [61] software packages were used for genome assembly. Annotation was performed by the NCBI Prokaryotic Genome Annotation Pipeline (https://www.ncbi.nlm.nih.gov/genome/annotation_prok/). An additional 46 genomes, including ten of *S. argenteus*, six of *S. schweitzeri*, and 30 of *S. aureus*, were obtained from the NCBI public database (ftp://ftp.ncbi.nlm.nih.gov/genomes/refseq/bacteria/Staphylococcus_aureus/) and Whole Genome Shotgun (WGS) sequencing projects databases (http://www.ncbi.nlm.nih.gov/Traces/wgs/) in May 2016. The genomic data was generated from the genome sequences and related files from NCBI. The STs were assigned by the public MLST database of *S. aureus* (http://saureus.mlst.net/), using the seven homologous fragments from the genome sequences.

### Assignment of homologous gene families

The deduced amino acid sequences of all CDSs from the 51 genomes were adjusted to a prescribed format and were grouped into homologous gene families using OrthoMCL version 2.0.9 [62] based on sequence similarity. The BLAST reciprocal best hit algorithm [63] was employed with a 70% match cutoff and 1e-5 e-value cutoff, and Markov Cluster Algorithms (MCL) [64] were applied with an inflation index of 1.5.

To account for pseudogenes and cases where genes were missed in the gene-calling step, BLAST (blast + package, version 2.2.29) [65] was used to align all genes of one family (assigned by OrthoMCL) against the genome sequence of the strains in which the family was not previously identified. If an alignment with at least 90% nucleotide sequence identity covering at least 90% of the sequence length was detected in the genome, the gene/pseudogene was considered present and the related location was recorded in the complete homologous gene families list using a different font (Additional file 3: Table S1). This produced a more robust pan-genome outcome and accommodated the differences in the gene prediction. For example, 137 and 140 genes were newly detected in genomes of strains *S. aureus* DAR4 and RKI4, respectively. The sizes of the four components of the pan-genome (CR, CV, VR and UQ) were simulated as has been done in previous studies [66] using Perl scripts. For example, pan-genome analysis of SAC was conducted starting from one single genome to 51 genomes. Genomes were added 1000 times in a randomized order without replacement for each fixed number of genomes, and the gene reservoir was accumulated. The functional category of each homologous gene family was determined by using the BLAST program locally or on the web server against the Cluster of Orthologous Groups (COGs), UniProt, KEGG Automatic Annotation Server (KAAS), and ACLAME database [27, 38, 39, 53], with 50% identity cutoff and 1e-5 e-value.

### Phylogenetic analysis and bANI

To determine the phylogenetic relationships of SAC members based on genomic data, both supermatrix and gene content methods were applied to infer phylogenetic trees. For the supermatrix approach, 1375 single-copy orthologous genes shared by all 51 SAC strains and *S. simiae* CCM 7213 (AEUN00000000.1) were selected from the homologous gene families. For each orthologous family, protein sequences were aligned using Clustal Omega version 1.2.1 [67] and the resulting alignments of individual proteins were concatenated to infer the phylogeny using the maximum likelihood algorithm (ML) in RAxML version 8.1.2 [68] under the substitution matrix JTTDCMUT which was selected by the Perl script in the software package. The gene content matrix was parsed for the gene content method using a phyletic pattern indicating the presence (1) or absence (0) of the respective non-core genes of all strains. The Neighbor-Joining distance between pairwise genomes was calculated based on the gene content matrix to reconstruct the gene content dendrogram using package Splits Tree version 4.13.1 [69].

The SNPs among *S. argenteus* ST2250 strains were detected, extracted and concatenated from nucleotide sequence alignments of 2348 orthologous gene families using Perl scripts. The maximum likelihood algorithm (ML) in RAxML version 8.1.2 [68] was used to infer the phylogeny under the substitution matrix GTR. The Neighbor-Joining phylogenetic trees of 16 genes of capsular polysaccharide (CP) were constructed based on amino acid sequence using the Poisson correction method in MEGA6 [70]. The bANI tests were simulated as done in previous studies [26]. Briefly, the core genes’ nucleotide sequences of each of the genomes were extracted and used in BLAST against the other whole genome sequences, and all the identity values were grouped into six groups, including three intraspecific groups and three interspecific groups. Then the data of each group were counted and presented as percentages in a histogram.

### Population structure

To investigate the population structure of the SAC and the relationships among species groups, the Markov chain Monte Carlo (MCMC) based program Structure version 2.3.4 [36] was used to cluster individuals into populations. Initially, we treated core genes as MLST sequence data from Extended FASTA Format into the Structure Format using xmfa2struct (available from http://www.xavierdidelot.xtreemhost.com/clonalframe.htm). The admixture ancestry model with the assumption of correlated allele frequencies among populations was used. We ran the simulation five times under a burn-in period of 10,000 and a run length of 20,000 MCMC, without prior population information. K values from one to seven were tested to identify the best K value, represented by the highest value of K and ΔK [36]. Results of the five independent runs were averaged for each K value was used to determine the most likely model, i.e., the one with the highest likelihood, and they were subsequently plotted using Distruct version 1.1 [71]. The identification of the best K was evaluated following the ΔK-method through local program Structure Harvester [37].

### Species-specific genes and virulence genes

Species-specific genes were inferred from the homologous gene families list (Additional file 3: Table S1) and reanalyzed against the missing species genome using BLAST algorithm to confirm that they were lost or became pseudogenes. The 111 virulence genes were identified and collected from a virulence factor database (VRprofile, http://bioinfo-mml.sjtu.edu.cn/VRprofile/) [72] and a recent review of the literature [45]. Reference sequences of these genes were used with BLAST algorithm to find the homologous gene families presented in Additional file 3: Table S1. Some virulence genes, which have a paralogous relationship and may be assigned to one gene family by OrthoMcl software (Additional file 9: Table S2), were separated for subsequent analysis. The pairwise k-tuple distance among each of these virulence genes was calculated and outputted from the Clustal Omega (version 1.2.1) created alignment [67]. The distance matrix was then parsed using Perl script. Principal component analysis (PCA) were performed using software Past software version 3.13 (http://folk.uio.no/ohammer/past/), and other statistical analyses were performed using the R package (version 3.1.1, http://www.R-project.org).

## Additional files

Additional file 1: Figure S1. Phylogenetic distribution of the 30 *S. aureus* strains used used in this study. This neighbor-joining phylogenetic tree was constructed based on the predicted amino acid sequences of 3103 STs currently available in MLST database (http://saureus.mlst.net/). The colored dots represent the STs of *S. aureus* strains used in this study. A red dot represents one ST while each green dot represents two closely related STs. (PDF 386 kb)

Additional file 2: Figure S2. Comparison of CDS number, genome size and GC content among SAC members. Additional genomic and typing information are shown in Fig. 1. (PDF 127 kb)

Additional file 3: Table S1. Complete list of homologous gene families in *S. aureus* complex (SAC) genomes. Fifty-one SAC genomes were analyzed. The 4249 homologous gene families were identified and are listed. Several gene loci were found to be missing in gene calling or present as pseudogenes. Notations are as follows: red text, internal stop; bold text, frameshifted; italicized text, incomplete because of assembly; underlined text, multiple problems; italicized and underlined, missing start or stop codon; regular, missing in original gene calling. (XLSX 1666 kb)

Additional file 4: Figure S3. Pan-genome features and related COG functional categories of SAC and *S. aureus*. The *S. aureus* and SAC pan-genomes were grouped into four categories: UQ, unique genes; VR, variable genes; CV, core-variable genes; CR, core genes. (a & b): The number of genes in each pan-genome category (y-axis) for a given number of genomes (x-axis) was computed and is presented for (a) SAC and (b) *S. aureus*. The upper and lower edges of the boxes indicate the 25th and 75th percentiles, respectively, and the horizontal black lines indicate the 50th percentile for 1000 computations where the order of genome input was random. Hollow dots represent abnormal values. Regression analysis of the four SAC curves fitted to the following functions: CR, P(*N*) = −211.1ln(*N*) + 2539.4, *R* ^*2*^ = 0.9814; CR + CV, P(*N*) = 2445.6 *N* ^-0.051^, *R* ^*2*^ = 0.9365; CR + CV + VR, P(*N*) = 247.53ln(*N*) + 2733.6, *R* ^*2*^ = 0.9638; CR + CV + VR + UQ, P(*N*) = 2604.5 *N* ^0.1232^, *R* ^*2*^ = 0.9995. Regression analysis of four *S. aureus* curves fitted functions as follows: CR, P(*N*) = −246.4ln(*N*) + 2616.7, *R* ^*2*^ = 0.9932; CR + CV, P(*N*) = 2571 *N* ^-0.062^, *R* ^*2*^ = 0.9696; CR + CV + VR, P(*N*) = 211.25ln(*N*) + 2755.1, *R* ^*2*^ = 0.9572; CR + CV + VR + UQ, P(*N*) = 2654.4 *N* ^0.1164^, *R* ^*2*^ = 0.9994. (c & d) The number of genes in each pan-genome group (UQ, VR, CV, or CR) was determined for each functional group (Cluster of Orthologous Groups, or COGs) for both (c) SAC and (d) *S. aureus*. COG codes: C, Energy production and conversion; D, Cell cycle control, cell division; E, Amino acid transport and metabolism; F, Nucleotide transport and metabolism; G, Carbohydrate transport and metabolism; H, Coenzyme transport and metabolism; I, Lipid transport and metabolism; J, Translation, ribosomal structure and biogenesis; K, Transcription; L, Replication, recombination and repair; M, Cell wall/membrane/envelope biogenesis; N, Cell motility; O, Posttranslational modification, protein turnover, chaperones; P, Inorganic ion transport and metabolism; Q, Secondary metabolites biosynthesis, transport and catabolism; R, General function prediction only; S, Function unknown; T, Signal transduction mechanisms; U, Intracellular trafficking, secretion, and vesicular transport; V, Defense mechanisms; W, Extracellular structures; X, Mobilome, prophages, transposons. (PDF 371 kb)

Additional file 5: Figure S4. Pan-genome features and related COG functional categories of *S. argenteus* and *S. schweitzeri*. The *S. argenteus* and *S. schweitzeri* pan-genomes were grouped into four categories: UQ, unique genes; VR, variable genes; CV, core-variable genes; CR, core genes. (a & b) The number of genes in each pan-genome category (y-axis) for a given number of genomes (x-axis) was computed and is presented for (a) *S. argenteus* and (b) *S. schweitzeri*. The upper and lower edges of the boxes indicate the 25th and 75th percentiles, respectively, and the horizontal black lines indicate 50th percentile under 1000 computations where the order of genome input was random. Hollow dots represent abnormal values. Regression analysis of the four *S. argenteus* curves fitted to the following functions: CR, P(*N*) = 2498.1 *N* ^-0.048^, *R* ^*2*^ = 0.9883; CR + CV, P(*N*) = 2499 *N* ^-0.039^, *R* ^*2*^ = 0.9644; CR + CV + VR, P(*N*) = 114.97ln(*N*) + 2540.3, *R* ^*2*^ = 0.9783; CR + CV + VR + UQ, P(*N*) = 2506.1 *N* ^0.0752^, *R* ^*2*^ = 0.9975. Regression analysis of four *S. schweitzeri* curves fitted functions as follows: CR, P(*N*) = 2483 *N* ^-0.064^, *R* ^*2*^ = 0.9757; CR + CV, P(*N*) = 2490.8 *N* ^-0.058^, *R* ^*2*^ = 0.9666; CR + CV + VR, P(*N*) = −11.771 *N* ^2^ + 116.17 *N* + 2379.7, *R* ^*2*^ = 0.9861; CR + CV + VR + UQ, P(*N*) = 209.05ln(*N*) + 2509.8, *R* ^*2*^ = 0.9999. . (c & d) The number of genes in each pan-genome group (UQ, VR, CV, or CR) was determined for each functional group (Cluster of Orthologous Groups, or COGs) for both (c) *S. argenteus* and (d) *S. schweitzeri*. COG codes are the same as the described in the legend to Additional file 5: Figure S4. (PDF 276 kb)

Additional file 6: Figure S5. A Venn Diagram showing the pan-genome categories that are common among and shared between species within the SAC. Sharing gene families contain at least one genome of the species of interest. Core and core-variable gene families are refer to core gene (CR) and core-variable gene (VR) as described in the main text, respectively. (PDF 138 kb)

Additional file 7: Figure S6. Phylogenetic relatedness of SAC species based on predicted amino acid sequences of genes for *agr* signaling pathway. Phylogenetic trees were constructed using the Neighbor-Joining method in order to infer evolutionary history and relatedness for SAC species. The evolutionary distances were computed using the Poisson correction method. Genes from *S. aureus*, *S. argenteus*, and *S. schweitzeri* are represented by red, green, and blue dots, respectively. (PDF 56 kb)

Additional file 8: Figure S7. Phylogenetic relatedness of SAC species based on predicted amino acid sequences of genes for synthesis of capsular polysaccharide. Phylogenetic trees were constructed using the Neighbor-Joining method in order to infer evolutionary history and relatedness for SAC species. The evolutionary distances were computed using the Poisson correction method. For each tree the bar indicates 0.005 substitutions per site. Genes from *S. aureus*, *S. argenteus*, and *S. schweitzeri* are represented by red, green, and blue dots, respectively. For the trees based *capH-K*, *S. aureus* strains of serotype 5 and serotype 8 are shaded in light or dark gray, respectively. (PDF 262 kb)

Additional file 9: Table S2. Prevalence and relatedness of virulence genes among *S. aureus* complex (SAC) genomes. The presence/absence and interspecies divergence was analyzed for 111 virulence genes from 51 SAC genomes (30 *S. aureus*, 15 *S. argenteus* and 6 *S. schweitzeri*. Five virulence genes, *sed*, *see*, *sej*, *selr* and *etb*, are not listed in the table because they were not detected in any of the 51 SAC genomes examined. ^a^ *, these genes have tandem repeat regions, so the interspecies divergence of the nucleotide sequences may not clearly reflect the true evolutionary divergence. ^b^ The number of SAC genomes of each species harboring each virulence gene. ^c^ The gene family numbers as listed in Additional file 3: Table S1. ^d^ NA, not applicable; SA, *S. aureus*; SG, *S. argenteus*; SW, *S. schweitzeri*. ^e^ Maximum likelihood phylogenetic trees were contructed based on nucleotide sequence, using substitution matrix GTR and executing 100 rapid bootstrap inferences. The species names were omitted in tip labels. ^f^ p.g., pseudogene. (XLSX 1797 kb)

## Abbreviations

AIP: Autoinducing peptide
AU: Australia
bANI: Average nucleotide identities based on BLAST
CA-MRSA: Community-acquired methicillin-resistant *S. aureus*
CC: Coding sequence
CDS: Clonal complex
CN: China
COG: Cluster of Orthologous Groups
CoNS: Coagulase-negative staphylococci
CP: Capsular polysaccharide
CR: Core genes
CRISPR-Cas: Clustered regularly interspaced short palindromic repeats-CRISPR-associated proteins
CrtN: Diapophytoene desaturases
CV: Core variable genes
HGT: Horizontal gene transfer
KAAS: KEGG Automatic Annotation Server
MCL: Markov Cluster Algorithms
MCMC: Markov chain Monte Carlo
MGE: Mobile genetic elements
ML: Maximum Likelihood
MLST: Multilocus sequence typing
MRSA: Methicillin-resistant *S. aureus*
PCA: Principal component analysis
PVL: Panton-Valentine leukocidin
QS: Quorum sensing system
SAC: *S. aureus* complex
SCC*mec*: Staphylococcal cassette chromosome *mec* element
SNP: Single nucleotide polymorphism
SRA: Sequence Read Archive
ST: Sequence type
UQ: Unique gene
US: United States of America
VR: Variable genes
WGS: Whole Genome Shotgun
