# Supplementary figures and images for: Preliminary comparative genomics revealed pathogenic potential and international spread of Staphylococcus argenteus
Source: BMC Genomics. 2017 Oct 23;18:808. doi: 10.1186/s12864-017-4149-9 (PMC5651615; doi:10.1186/s12864-017-4149-9)

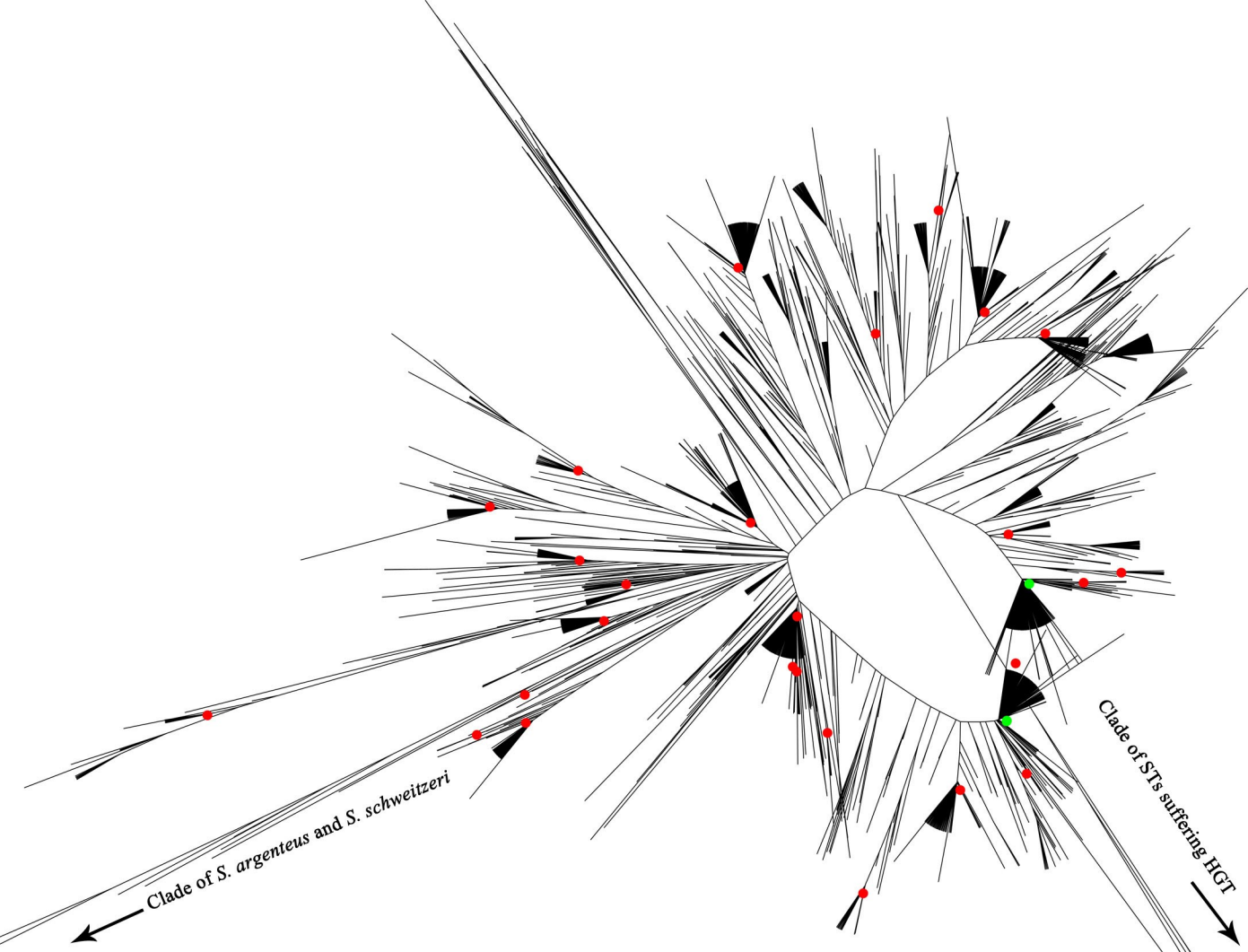

Supplement: Supplementary file 1 — Phylogenetic distribution of the 30 S. aureus strains used used in this study. This neighbor-joining phylogenetic tree was constructed based on the predicted amino acid sequences of 3103 STs currently available in MLST database (http://saureus.mlst.net/). The colored dots represent the STs of S. aureus strains used in this study. A red dot represents one ST while each green dot represents two closely related STs. (PDF 386 kb) [file 12864_2017_4149_MOESM1_ESM.pdf]

CDS number

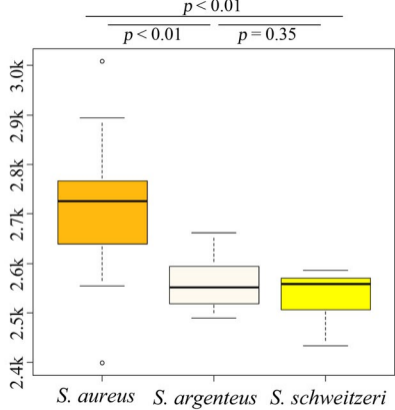

Genome size (Mb)

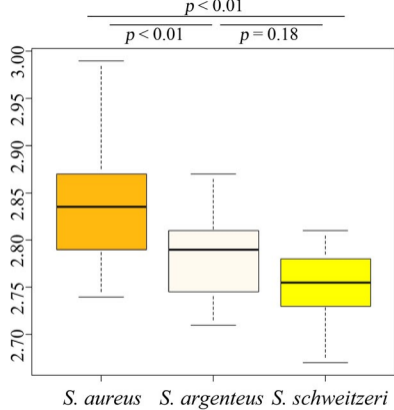

GC content (%)

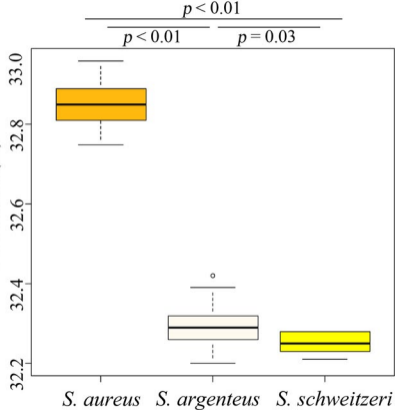

Supplement: Supplementary file 2 — Comparison of CDS number, genome size and GC content among SAC members. Additional genomic and typing information are shown in Fig. 1. (PDF 127 kb) [file 12864_2017_4149_MOESM2_ESM.pdf]

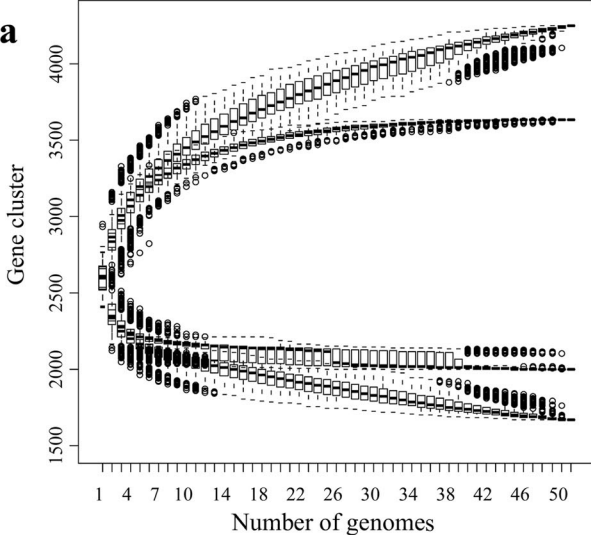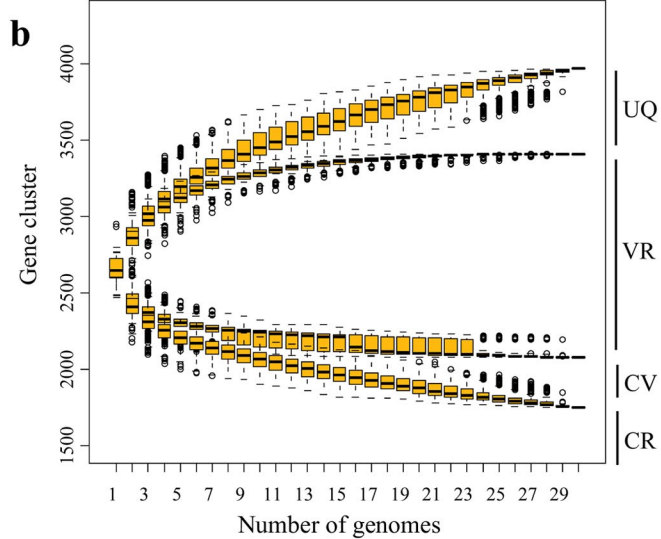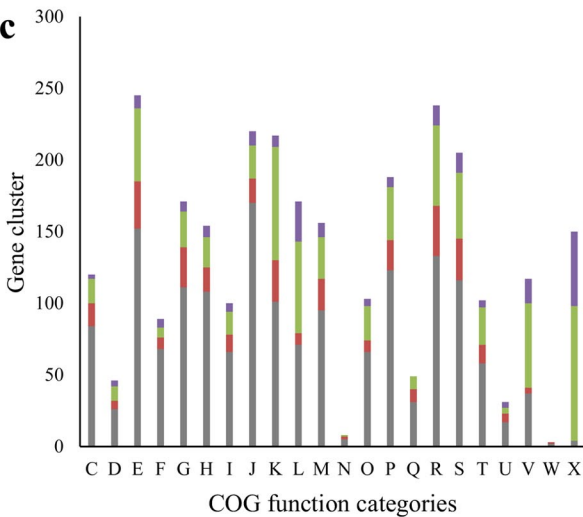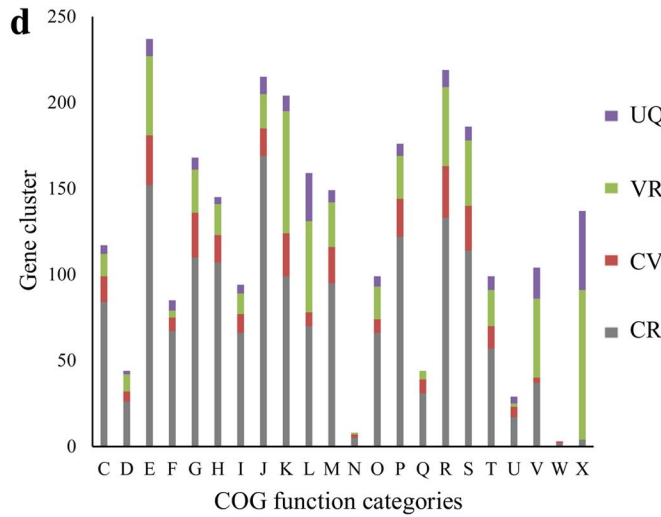

Supplement: Supplementary file 4 — Pan-genome features and related COG functional categories of SAC and S. aureus. The S. aureus and SAC pan-genomes were grouped into four categories: UQ, unique genes; VR, variable genes; CV, core-variable genes; CR, core genes. (a & b): The number of genes in each pan-genome category (y-axis) for a given number of genomes (x-axis) was computed and is presented for (a) SAC and (b) S. aureus. The upper and lower edges of the boxes indicate the 25th and 75th percentiles, respectively, and the horizontal black lines indicate the 50th percentile for 1000 computations where the order of genome input was random. Hollow dots represent abnormal values. Regression analysis of the four SAC curves fitted to the following functions: CR, P(N) = −211.1ln(N) + 2539.4, R 2 = 0.9814; CR + CV, P(N) = 2445.6 N -0.051, R 2 = 0.9365; CR + CV + VR, P(N) = 247.53ln(N) + 2733.6, R 2 = 0.9638; CR + CV + VR + UQ, P(N) = 2604.5 N 0.1232, R 2 = 0.9995. Regression analysis of four S. aureus curves fitted functions as follows: CR, P(N) = −246.4ln(N) + 2616.7, R 2 = 0.9932; CR + CV, P(N) = 2571 N -0.062, R 2 = 0.9696; CR + CV + VR, P(N) = 211.25ln(N) + 2755.1, R 2 = 0.9572; CR + CV + VR + UQ, P(N) = 2654.4 N 0.1164, R 2 = 0.9994. (c & d) The number of genes in each pan-genome group (UQ, VR, CV, or CR) was determined for each functional group (Cluster of Orthologous Groups, or COGs) for both (c) SAC and (d) S. aureus. COG codes: C, Energy production and conversion; D, Cell cycle control, cell division; E, Amino acid transport and metabolism; F, Nucleotide transport and metabolism; G, Carbohydrate transport and metabolism; H, Coenzyme transport and metabolism; I, Lipid transport and metabolism; J, Translation, ribosomal structure and biogenesis; K, Transcription; L, Replication, recombination and repair; M, Cell wall/membrane/envelope biogenesis; N, Cell motility; O, Posttranslational modification, protein turnover, chaperones; P, Inorganic ion transport and metabolism; Q, Secondary metabolites bios [file 12864_2017_4149_MOESM4_ESM.pdf]

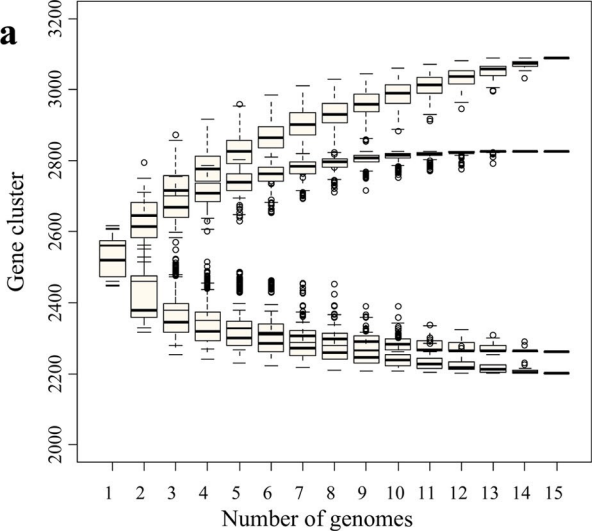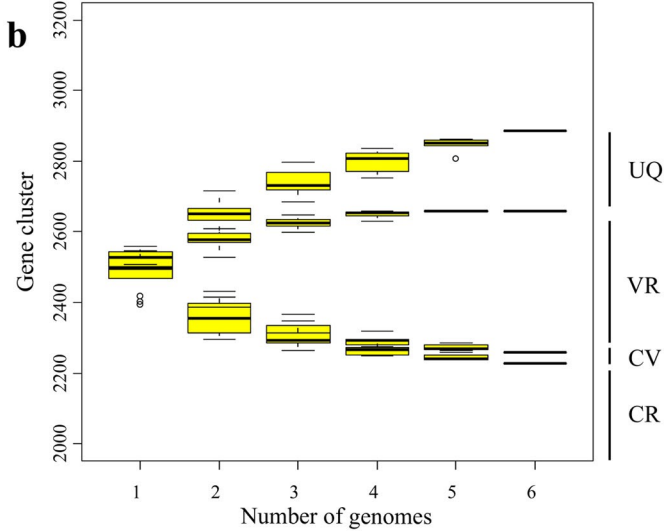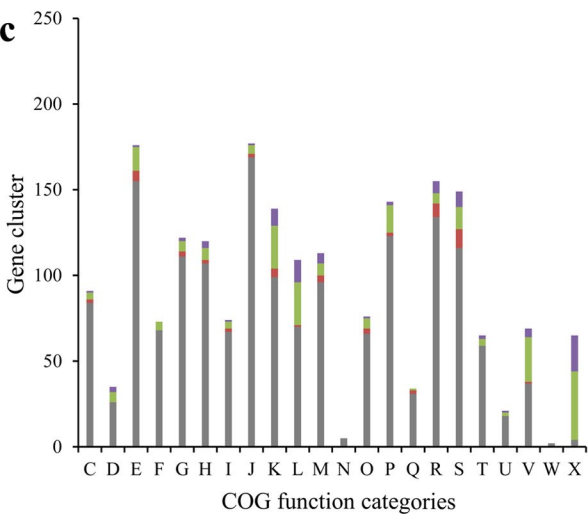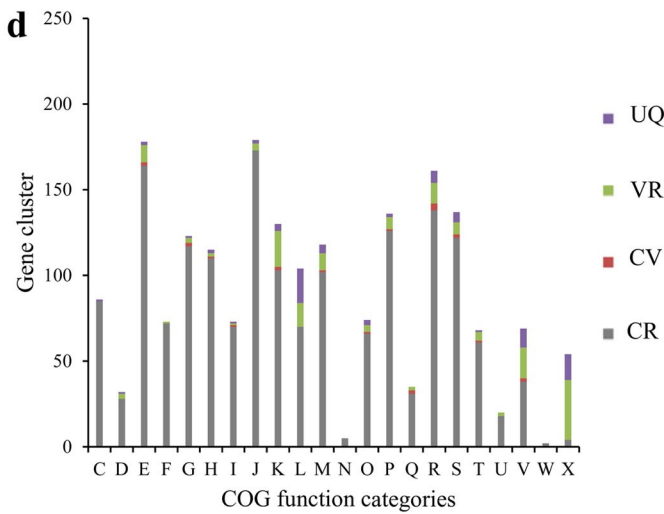

Supplement: Supplementary file 5 — Pan-genome features and related COG functional categories of S. argenteus and S. schweitzeri. The S. argenteus and S. schweitzeri pan-genomes were grouped into four categories: UQ, unique genes; VR, variable genes; CV, core-variable genes; CR, core genes. (a & b) The number of genes in each pan-genome category (y-axis) for a given number of genomes (x-axis) was computed and is presented for (a) S. argenteus and (b) S. schweitzeri. The upper and lower edges of the boxes indicate the 25th and 75th percentiles, respectively, and the horizontal black lines indicate 50th percentile under 1000 computations where the order of genome input was random. Hollow dots represent abnormal values. Regression analysis of the four S. argenteus curves fitted to the following functions: CR, P(N) = 2498.1 N -0.048, R 2 = 0.9883; CR + CV, P(N) = 2499 N -0.039, R 2 = 0.9644; CR + CV + VR, P(N) = 114.97ln(N) + 2540.3, R 2 = 0.9783; CR + CV + VR + UQ, P(N) = 2506.1 N 0.0752, R 2 = 0.9975. Regression analysis of four S. schweitzeri curves fitted functions as follows: CR, P(N) = 2483 N -0.064, R 2 = 0.9757; CR + CV, P(N) = 2490.8 N -0.058, R 2 = 0.9666; CR + CV + VR, P(N) = −11.771 N 2 + 116.17 N + 2379.7, R 2 = 0.9861; CR + CV + VR + UQ, P(N) = 209.05ln(N) + 2509.8, R 2 = 0.9999. . (c & d) The number of genes in each pan-genome group (UQ, VR, CV, or CR) was determined for each functional group (Cluster of Orthologous Groups, or COGs) for both (c) S. argenteus and (d) S. schweitzeri. COG codes are the same as the described in the legend to Additional file 5: Figure S4. (PDF 276 kb) [file 12864_2017_4149_MOESM5_ESM.pdf]

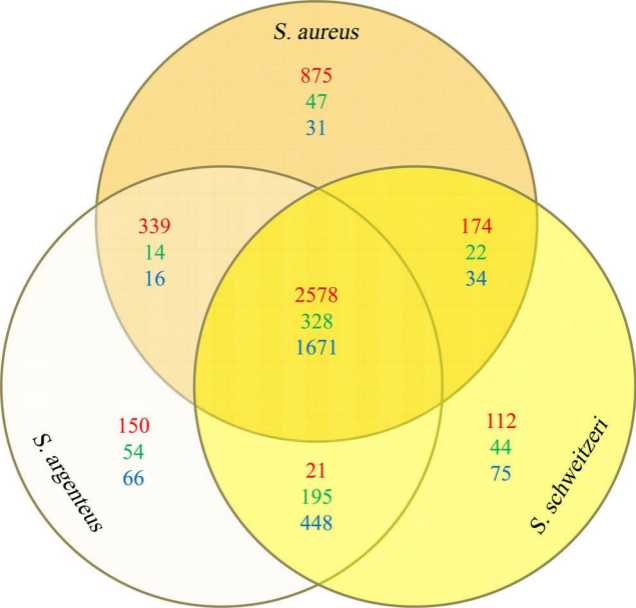

- Sharing gene families
- Core-variable gene families
- Core gene families

Supplement: Supplementary file 6 — A Venn Diagram showing the pan-genome categories that are common among and shared between species within the SAC. Sharing gene families contain at least one genome of the species of interest. Core and core-variable gene families are refer to core gene (CR) and core-variable gene (VR) as described in the main text, respectively. (PDF 138 kb) [file 12864_2017_4149_MOESM6_ESM.pdf]

**a**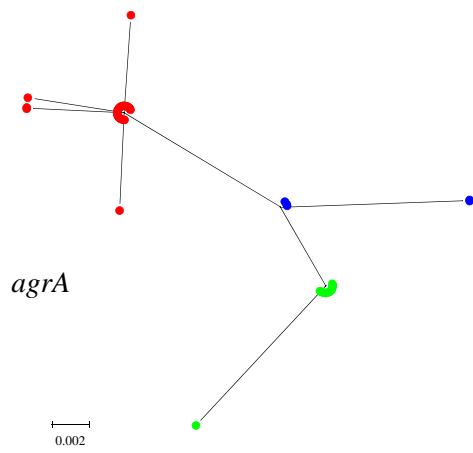**b**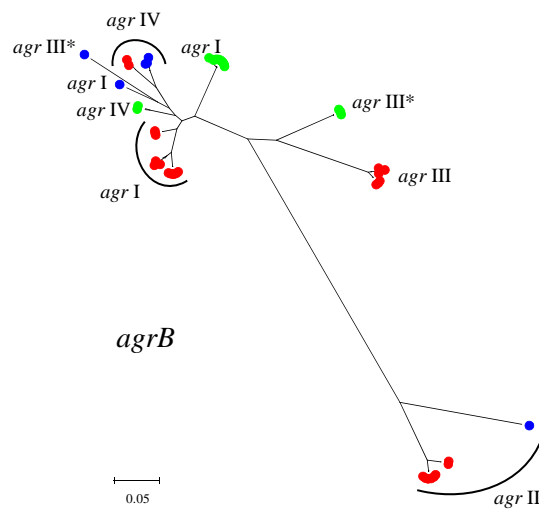**c**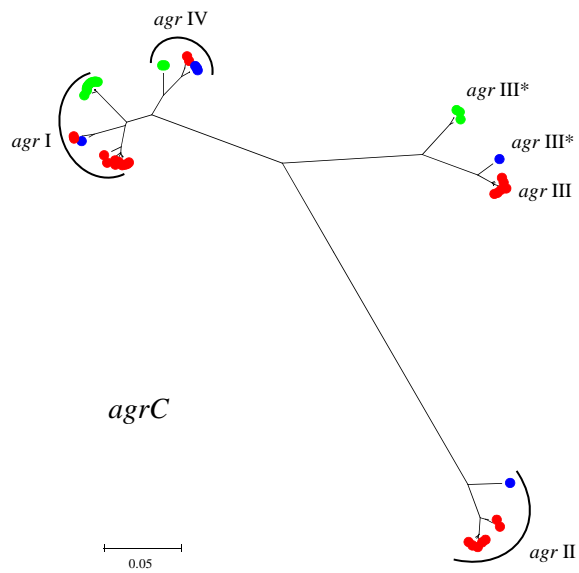**d**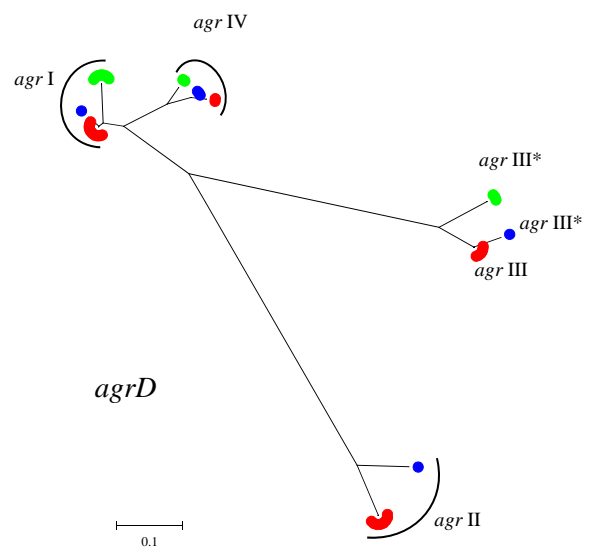

Supplement: Supplementary file 7 — Phylogenetic relatedness of SAC species based on predicted amino acid sequences of genes for agr signaling pathway. Phylogenetic trees were constructed using the Neighbor-Joining method in order to infer evolutionary history and relatedness for SAC species. The evolutionary distances were computed using the Poisson correction method. Genes from S. aureus, S. argenteus, and S. schweitzeri are represented by red, green, and blue dots, respectively. (PDF 56 kb) [file 12864_2017_4149_MOESM7_ESM.pdf]

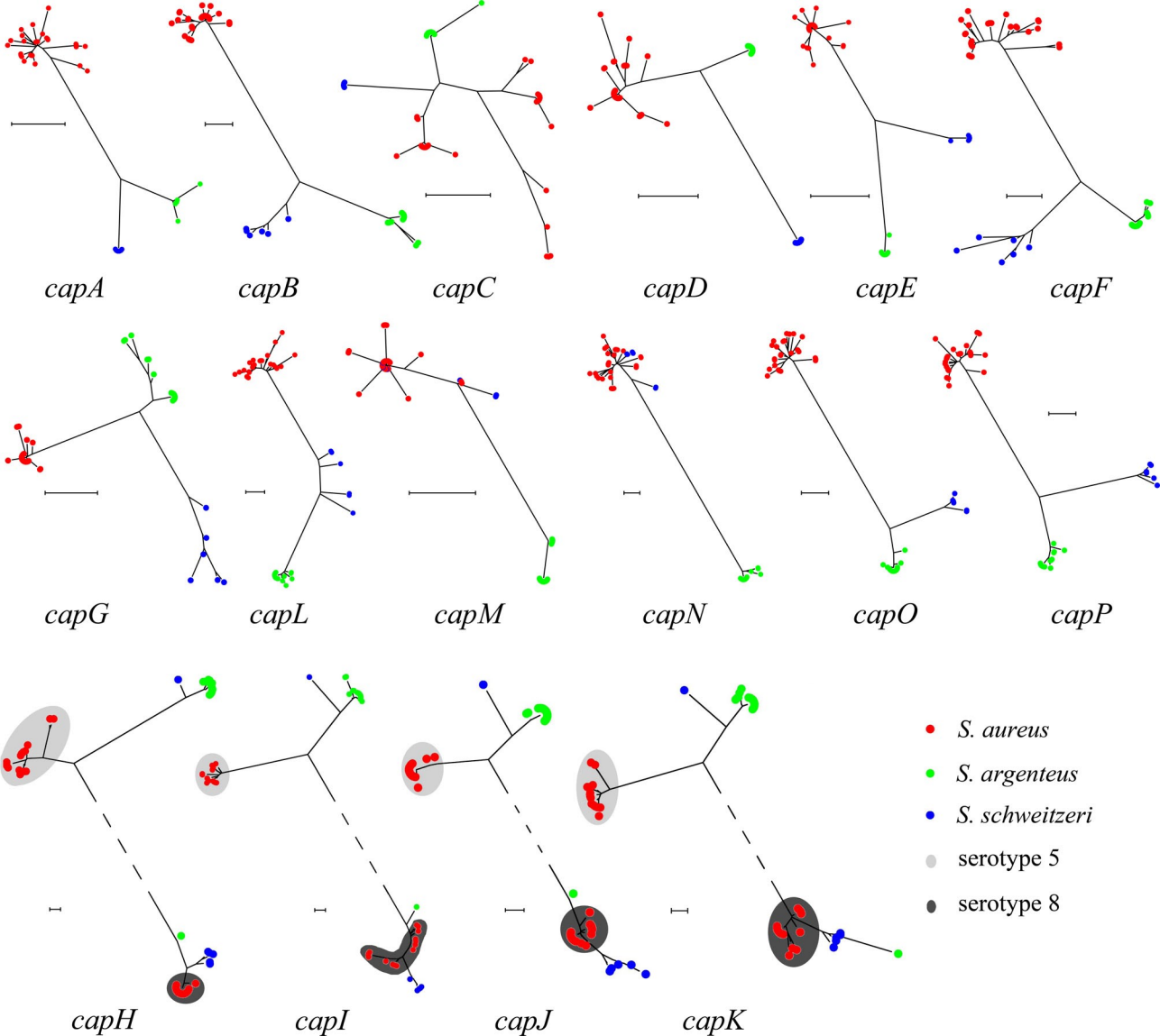

Supplement: Supplementary file 8 — Phylogenetic relatedness of SAC species based on predicted amino acid sequences of genes for synthesis of capsular polysaccharide. Phylogenetic trees were constructed using the Neighbor-Joining method in order to infer evolutionary history and relatedness for SAC species. The evolutionary distances were computed using the Poisson correction method. For each tree the bar indicates 0.005 substitutions per site. Genes from S. aureus, S. argenteus, and S. schweitzeri are represented by red, green, and blue dots, respectively. For the trees based capH-K, S. aureus strains of serotype 5 and serotype 8 are shaded in light or dark gray, respectively. (PDF 262 kb) [file 12864_2017_4149_MOESM8_ESM.pdf]
